# Supplementation of high levels of essential fatty acids using soybean oil in lactation diets benefits the subsequent reproduction of sows but can be detrimental to the performance of young sows if provided after weaning

**DOI:** 10.1186/s40104-025-01192-y

**Published:** 2025-04-10

**Authors:** Garrin Lee Shipman, David Rosero, Eric van Heugten

**Affiliations:** 1 Department of Animal Science, North Carolina State University, Raleigh, NC 27695 USA; 2 The Hanor Company, Inc., Enid, OK 73701 USA

**Keywords:** α-Linolenic acid, Lactation, Linoleic acid, Sows, Subsequent reproduction, Wean-to-breeding

## Abstract

**Background:**

This study investigated the potential impacts of increasing linoleic and α-linolenic acid intake during lactation and wean-to-breeding on subsequent reproduction of sows. A total of 309 sows (PIC Camborough L42) were balanced by parity (140 and 169 sows representing parity 1 to 2 [P1-2] and 3 to 9 [P3+], respectively) and assigned within parity to a 2 × 2 factorial arrangement. Factors included essential fatty acid (EFA) supplementation (control diets containing 1.2% linoleic and 0.15% α-linolenic acid or diets with 3.0% linoleic and 0.38% α-linolenic acid) and supplementation period (lactation or wean-to-breeding). Tallow (low EFA diets) or soybean oil (high EFA diets) were included at 4% in sorghum-soybean meal-wheat middlings-based diets to attain targeted EFA levels.

**Results:**

High levels of EFA fed during lactation had no effect on feed intake or litter performance, but increased subsequent farrowing rate (*P* = 0.027; 82.1% vs. 70.4%), tended to reduce the proportion of sows removed (*P* = 0.070; 12.4% vs. 20.8%), decreased the number of total pigs born in the following litter (*P* = 0.072; 15.3 vs. 16.2), and increased total pigs born alive per 100 sows weaned (*P* = 0.062; 1,122 vs. 974), regardless of sow parity. Young sows (P1-2) consuming the high EFA diet during lactation displayed a shorter wean-to-estrus interval (*P* = 0.035; 4.2 vs. 4.6), but P3+ sows were unaffected. Increasing EFA intake for P3+ sows, but not P1-2 sows, resulted in more sows bred by d 5 (*P* = 0.028; 91.1% vs. 81.7%) and more mummies in the subsequent litter (*P* = 0.040; 0.32 vs. 0.16). Feeding increased EFA to P1-2 sows during the wean-to-breeding period decreased subsequent farrowing rate (*P* = 0.042; 72.0% vs. 87.7%), and increased removal rate (*P* = 0.003; 28.8% vs. 9.4%). Total pigs born alive per 100 sows weaned was reduced (*P* = 0.007) in P1-2 sows when supplemented with EFA during wean-breeding (939 vs. 1,149) but was not impacted in P3+ sows (1,131 vs. 982).

**Conclusions:**

Supplemental EFA in lactation diets benefited subsequent reproduction of sows, regardless of parity. Increasing dietary levels of EFA during the wean-to-breeding period to younger sows negatively impacted subsequent reproduction.

## Background

Excessive mobilization of body tissues during lactation is a contributor to reproductive inefficiency, including extended wean-to-estrus interval (WEI), failure to display estrous cycles, or inability to maintain pregnancy [1]. In commercial production, reproductive inefficiency ranks as the major reason for culling sows, followed by locomotive issues and old age [2, 3]. Increasing the intake of dietary n-6 and n-3 fatty acids during lactation has been shown to have beneficial impacts on subsequent reproductive performance [4–6]. Linoleic acid (C18:2n6) and α-linolenic acid (C18:3n3) are essential fatty acids (EFA) and precursors to prostanoids that have key roles in reproductive functions. As such, supplementation of EFA has been suggested to support reproduction through a combination of hormonal regulation (prostaglandins and progesterone), improved follicular and embryonic development, and enhanced post-partum recovery [7–9]. Essential fatty acids must be sourced through the diet because mammals lack the enzymatic capability to insert a double bond past the delta-10 carbon [10]. Essential fatty acids are also secreted in milk to stimulate growth in neonatal pigs. Entering a negative EFA balance and mobilization of EFA from body tissues results in compromised subsequent reproductive performance that can be improved through supplementation, especially in mature sows [11].

Increased linoleic acid intake (> 115 g linoleic acid/d) during lactation resulted in a higher number of sows being weaned, increased proportion of sows returning to breeding, increased pregnancy retention rates, a greater number pigs born in the subsequent litter, as well as a reduced number of pigs born dead or mummified [5, 6]. Inclusion of at least 0.45% α-linolenic acid in conjunction with elevated linoleic acid resulted in a higher number of sows being bred following weaning and maintaining the highest percentage of pregnancies [5]. Subsequent litter size appeared not to be influenced in response to varying α-linolenic acid levels. More importantly, control sows fed low levels of both EFA (dietary inclusions of < 2.1% linoleic acid and < 0.15% α-linolenic acid) in lactation had greater cull rates and reduced subsequent farrowing rates [5]. In swine diets, supplemental lipids have traditionally been used to provide energy, but more recently their value as a source of EFA has been recognized [12, 13]. The concentrations of EFA varies among lipid sources, in which lower concentrations of EFA are found in animal fats, while plant oils contain greater concentrations of EFA. From a practical perspective, tallow is very low in EFA, while soybean oil is considered the gold standard with significant concentrations of both linoleic and α-linolenic acid [10, 12].

The studies by Smits et al. [4], Rosero et al. [5], and van Wetterre [6] investigated EFA supplementation during lactation only, but not during the breeding or gestation periods. While it is important to improve nutrient intake of the modern lactating sow to meet her metabolic demands to nurse her rapidly growing litter [14], inadequate feeding during gestation can be detrimental to subsequent performance and result in poor sustainability at the production level [15]. The consumption of EFA during the period before insemination has been demonstrated to improve the reproductive output of beef cows [16]. Thus, the influence of EFA intake following lactation into the wean-to-breeding period warrants investigation in breeding sows housed under commercial conditions. The objective of the current study was to determine the impact of the practical supplementation of EFA from soybean oil during lactation and the breeding period on the subsequent reproduction of sows.

## Materials and methods

### Animals, experimental design, and dietary treatments

A total of 309 sows (Camborough L42, PIC, Hendersonville, TN, USA) were placed in the farrowing facility of a 2,600 sow commercial-research farm in Oklahoma, USA, during the summer months of July to September. Sows in this experiment were humanely treated following the practices outlined in the Guide for the Care and Use of Animals in Agricultural Research and Teaching [17]. There were a total of 140 young sows (parity 1 and 2 [P1-2]; average parity 1.2 ± 0.4) and 169 sows representing mature sows (parity 3 to 9 [P3+]; average parity 4.1 ± 1.4). The average body condition scores (BCS) assessed using a sow caliper [18] were 12.8 (± 1.9) and 12.4 (± 1.6) for the P1-2 and P3+ groups, respectively, at the time of placement. A sow caliper score of 12 to 15 represents a back angle of 125˚ to 132.5˚ and is considered ideal. A one-unit increase or decrease in the sow caliper score corresponded to a 2.5˚ degree increase or decrease in the angle of the sow’s back [18]. Sows were randomly assigned within parity group to a 2 × 2 factorial arrangement of treatments. Sows were assigned to the study in 7 groups of 40 to 47 sows with equal representation of parity and treatments within each group. Factors included essential fatty acid (EFA) supplementation level (low EFA diets containing 1.2% linoleic and 0.15% α-linolenic acid or high EFA diets with 3.0% linoleic and 0.38% α-linolenic acid) and supplementation period (lactation or wean-to-breeding).

Diets were sorghum-soybean meal-based with wheat middlings (12% in lactation and 25% for the weaning-to-breeding period) (Table 1). Sorghum was chosen as the grain source to avoid the high linoleic acid content of corn. All ingredients were representative of local ingredient availability at the time the study was conducted and diets were designed based least-cost formulation. All nutrients exceeded the NRC (2012) [19] requirements for lactating and gestating sows. Experimental diets were formulated to a constant nutrient to an effective metabolizable energy (ME) ratio, including amino acids, calcium, phosphorus, vitamins, and minerals. The standardized ileal digestible lysine/ME content was 3.12 g/Mcal and 1.66 g/Mcal for the lactation and gestation diets, respectively (Table 1). Thus, diets contained 1.05% and 0.56% standardized ileal digestible lysine for lactation and gestation diets respectively, meeting NRC [19] nutrient requirement estimates and practical feeding guidelines [14]. The targeted concentrations of EFA were obtained by supplementing either 4% of tallow for the low EFA diet or 4% of soybean oil for the high EFA diet. Added lipids were sprayed on after the pre-determined dry mix time for dry ingredients. Feed was manufactured in a commercial feed mill using current good manufacturing practices (Hanor Company, Enid, OK, USA) and presented to sows in pelleted form.

**Table 1:** Composition of experimental diets (as-fed basis)^a^

Period	Lactation		Wean-to-breed	
EFA inclusion	Low	High	Low	High
Ingredients, %				
Sorghum	64.06	64.06	64.95	64.95
Soybean meal (46% CP)	15.85	15.85	1.90	1.90
Wheat middlings	12.0	12.0	25.0	25.0
Tallow	4.15	-	4.10	-
Soybean oil	-	4.15	-	4.10
Limestone	1.13	1.13	1.35	1.35
Monocalcium phosphate (21% P)	0.63	0.63	0.69	0.69
L-Lysine·HCl (78.8%)	0.55	0.55	0.30	0.30
L-Threonine (99%)	0.23	0.23	0.11	0.11
L-Tryptophan (98.5%)	0.02	0.02	-	-
L-Methionine (99%)	0.11	0.11	0.06	0.06
Salt	0.40	0.40	0.43	0.43
Vitamin-trace mineral premix^b^	0.28	0.28	0.28	0.28
Potassium, magnesium sulfate^c^	-	-	0.50	0.50
Betaine (96%)	0.20	0.20	-	-
Anti-caking aid^d^	0.10	0.10	0.20	0.20
Choline chloride (60%)	0.13	0.13	0.13	0.13
Mycotoxin mitigant^e^	0.10	0.10	-	-
Enzyme blend^f^	0.04	0.04	0.04	0.04
Synthetic color^g^	0.05	0.05	-	-
Calculated composition, %				
ME, Mcal/kg	3.35	3.38	3.35	3.38
Crude protein	16.24	16.24	11.68	11.68
Total Lys	1.15	1.15	0.64	0.64
SID Lys	1.05	1.05	0.56	0.56
SID Lys/ME, g/Mcal	3.14	3.11	1.67	1.65
Calcium	0.82	0.82	0.88	0.88
Total phosphorus	0.53	0.53	0.58	0.58
Crude fat	6.96	6.96	7.14	7.14
Linoleic acid (18:2n-6)	1.01	3.00	1.06	3.03
α-Linolenic acid (18:3n-3)	0.01	0.36	0.11	0.36

^a^ Diets were formulated to exceed NRC (2012) [19] requirements. Diets consisted of a control diet and a diet with supplemental essential fatty acids (EFA)

^b^ Supplied per kg of complete diet: vitamin A, 8,378 IU; vitamin D_3_, 1,764 IU; vitamin E, 77 IU; vitamin K, 3.1 mg; vitamin B_12_, 0.033 mg; riboflavin, 8.8 mg; d-pantothenate, 26.5 mg; niacin, 22 mg; thiamine, 5.5 mg; pyridoxine, 3.1 mg; folic acid, 2.6 mg; biotin, 0.39 mg; Zn, 125 mg; Fe, 100 mg; Mn, 50 mg; Cu, 25.0 mg; I, 0.7 mg; Se, 0.3 mg; phytase, 476 FTU (Quantum Blue, AB Vista, Marlborough, UK), and chromium, 0.2 mg

^c^ Dynamate (Mosaic, Plymouth, MN, USA). Added as a laxative

^d^ Dry anti-caking aid and non-nutritive carrier (KALLSIL, Kemin Industries, Inc., Des Moines, IA, USA)

^e^ Additive to mitigate mycotoxin levels (Defusion Plus, Provimi, Lewisburg, OH, USA)

^f^ Supplied per kilogram of complete diet: phytase, 1,227 FTU (Quantum Blue, AB Vista, Marlborough, UK) and xylanase, 8,000 units (Porzyme, Danisco Animal Nutrition, Marlborough, UK)

^g^ Colored iron oxide. Added only to lactation diets to verify experimental treatment while sows were in farrowing

### Lactation period

Dietary treatments commenced once sows entered the farrowing room at approximately 110 d of gestation. Sows were fed 1.8 kg of feed once per day prior to parturition. After farrowing, treatment diets were delivered individually to sows 4 times per day, in excess of appetite, by an automated feeding system (DryExact Pro, Big Dutchman, Vechta, Germany). Lactation diets were color coded by mixing differently colored iron oxide into the 2 lactation treatment diets at the time of manufacturing for visual confirmation. The amount of feed delivered was recorded by the feeding system and was adjusted for individual sows based on previous feed intake. If feed refusal occurred during lactation, feed refusal was collected, air-dried, and dry weight was recorded. Feed intake was calculated from the difference between feed provided and feed refused. Sows had free access to water. During the months of July to September that the study was conducted, daily mean temperatures averaged 25.4 ± 1.6 °C.

Litter information collected included total number of pigs born, pigs born alive, stillborn pigs, mummies, and the number of pigs cross-fostered and weaned. Newborn pigs were visually inspected, and the smallest pigs in each litter were counted, weighed, and considered small pigs if they were equal or less than 1.27 kg of body weight. These pigs were considered to be of lower viability and at greater risk of pre-weaning mortality compared to their heavier littermates by this production system. These piglets remained with the sows as part of the litter. Piglet cross-fostering was done 18 to 24 h after farrowing to allow for colostrum intake from their own mothers. Cross-fostering was done within litters that were assigned to the same treatment and litter size was standardized to approximately equal numbers of pigs per sow. Handling, processing, and vaccination of piglets was performed according to the recommendations of licensed veterinarians and were identical for all litters. A subsample of litters (*n* = 107) was weighed after cross-fostering and at weaning to calculate average daily gain during lactation. Piglet mortality was recorded for each litter. Litter weight gain was calculated as the difference between the weight of the litter on the day of weaning and the weight of the litter after cross-fostering. Pigs that did not reach 3.62 kg of bodyweight at weaning were considered no-value pigs and they were counted as part of the number of pigs weaned. Pigs did not receive creep feed or milk replacer during the experiment. Weaning occurred at 21.0 ± 3.1 d of lactation.

### Subsequent reproductive performance

At weaning, sows were moved into the breeding barn according to their assigned treatment. Sows were again assessed for BCS using a sow caliper. The breeding row at the sow facilities was equipped with drop feeders (capacity of 3.62 kg) and had two independent feeding lines. One-half of the row received the control and the other half the supplemental post-weaning diet. All sows were offered 3.6 kg/d until insemination, and feed was dispensed twice per day. Sows had once daily fence-line contact with a boar to facilitate estrus detection. Sows were artificially inseminated upon exhibiting physical signs of the first estrus. Semen used for artificial insemination was pooled from boars of the same genetic line (PIC boar L337) that originated from the same boar stud. Insemination doses contained 3 × 10^9^ sperm cells in 80 mL extender (PreservXtra, ReproQuest, Fitchburg,WI, USA). Pooled semen was delivered to the farm three times per week, properly stored within 15.0 to 16.7 °C, and completely used within 3 d. Sows were inseminated with a single dose at first display of estrus and then a second dose 24 h later. Sows that did not return to estrus within 14 d after weaning were removed from the dietary treatments. These removed sows were considered when calculating the pig born index (pigs born per 100 sows weaned) but not used in the final analysis for subsequent litter size. After insemination, sows were fed a commercial gestation diet and feed drops were adjusted to dispense amounts of feed depending on visual assessment of BCS (BCS 1 = 3.6 kg/d; BCS 2 = 2.7 kg/d; BCS 3 = 2.0 kg/d; BCS 4–5 = 1.6 kg/d). Feed was delivered twice per day. At d 90 of gestation, sows with a BCS of 1–2, which received 0.7 kg/d of additional feed.

An ultrasonic scanner (VSS-700 EZ-Pregchecker, Veterinary Sales and Services, Stuart, FL, USA) was used on d 35 of gestation to check pregnancy status. After confirmation of pregnancy, sows were moved to a common gestation area and housed in individual stalls until 3 d before their expected farrowing dates. Sows were observed for vaginal discharges and had once daily fence-line contact with a boar to detect sows returning to estrus. Data collected after weaning included days to estrus, number of sows bred, the number of sows that maintained pregnancy throughout gestation, and the number of sows that farrowed. Sow removal from the study due to abortion, returning to estrus, being culled or found deceased during any period of the gestation period were recorded. Removal rate was defined as the proportion of sows removed from the study relative to the number of sows weaned. In addition to sow reproductive performance, subsequent litter information, including total number of pigs born, number of pigs born alive, stillborn pigs, and mummies was collected.

### Chemical analyses

Feed samples were collected at the feed mill on a weekly basis throughout the lactation and breeding period and pooled within lactation and gestation treatments. Analysis (Table 2) of the diets was conducted by the Agricultural Experiment Station Chemical Laboratories, University of Missouri (Columbia, MO, USA) using AOAC [20] procedures. Diets were analyzed for moisture (Method 934.15), crude protein (Method 990.03), ash (Method 942.05), and fatty acid profile, including linoleic and α-linolenic acid (Methods 996.06 and 965.49).

**Table 2:** Proximate and fatty acid analysis of diets fed during lactation and the post-weaning period to sows allotted to diets containing low or high inclusions of essential fatty acids (EFA)

Period	Lactation		Wean-to-breed	
EFA inclusion	Low	High	Low	High
Proximate analysis, %				
Crude protein	16.9	16.8	12.2	11.9
Dry matter	89.9	90.0	89.8	87.1
Ash	4.4	4.4	4.7	4.9
Fatty acids, % of total fat				
C16:0	19.1	12.9	18.8	12.5
C18:0	11.1	3.6	10.4	2.8
C18:1n9	30.2	24.3	29.8	24.8
C18:2n6	25.2	50.7	27.1	51.8
C18:3n3	1.9	5.6	1.9	4.8
C20:4n6	0.02	0.1	0.02	0.05
C20:5n3	-	-	-	-
C22:6n3	0.1	0.1	0.1	0.2
Total n-6	25.2	50.8	27.1	51.9
Total n-3	2.0	5.7	2.0	5.0
n-6:n-3 ratio	12.6	8.9	13.6	10.4
Other fatty acids^a^	9.0	2.3	8.7	2.5
Total fatty acids	96.7	99.6	96.8	99.5

^a^ Two percent or less of the following fatty acids: 15:0, 17:0, 17:1, 18:1n9t, 18:1n11c, 18:2n6t, 18:3n6, 20:0, 20:1n9, 20:2n7, 21:0, 22:00, 22:1n9, and 24:0

### Calculations and statistical analyses

The pigs born index was determined to attain a representative value of fully formed pigs born per 100 weaned sows that subsequently farrowed. The index was calculated by the multiplying farrowing rate (sows farrowed as a proportion of sows weaned) by the number of pigs born in the subsequent litter [13].

Data for sow and litter performance during lactation were analyzed using the MIXED procedure of SAS (SAS Inst. Inc., Cary, NC, USA). The model included EFA inclusion during lactation, parity group, and their interaction as the fixed effects. Group of sows (7 groups of 40 to 47 sows) that entered the farrowing room together in the same location and at the same time was used as the random effect in the model. Sow was used as the experimental unit.

The WEI and sizes of the subsequent litters were fitted to a mixed linear model using the MIXED procedure of SAS. The model included EFA inclusion in lactation, EFA inclusion in the weaning-to-breeding period, parity group, and their interactions as fixed effects. Sow group at placement was considered the random effect. The proportion of sows weaned, bred, farrowed, culled as well as the cumulative proportion of sows bred within 4 to 5 d post-weaning were treated as binary data. Binary data were fitted to a non-linear model using the GLIMMIX procedure of SAS with a logit link function. The model included EFA inclusion in lactation, EFA inclusion in the wean-to-breeding period, parity group, and their interactions as fixed effects. Sows were retained in the same group throughout the subsequent breeding and gestation period, therefore, sow group at placement was used as the random effect. Probability values of < 0.05 were considered statistically significant and values between 0.05 and 0.10 were considered tendencies. A Tukey test was applied for multiple comparisons when differences were detected. A significant difference was observed for lactation length between dietary treatments. The impact of using lactation length as a covariate on outcome variables was tested and found to be not significant; therefore, lactation length was not included in the final model as a covariate. Values are presented as least squares means and standard error of the mean (SEM).

## Results

The total dietary concentrations of linoleic acid in the lactation and the post-weaning EFA supplemented diets were approximately 2 times that of the control diets and were approximately 2.5 to 3 times greater for α-linolenic acid (Table 2). This is consistent with expected concentrations based on the experimental design.

### Lactation period

No two-way interactions (*P* > 0.10) between parity group and EFA inclusion level during lactation were detected for any of the variables collected. Sows consuming high levels of EFA during lactation had shorter lengths of lactation (*P* = 0.020) than sows fed the low EFA diet in lactation (Table 3). Mature sows (P3+) tended to have shorter lactation lengths (*P* = 0.066) and significantly greater feed intake (*P* = 0.027) compared to P1-2 sows. The BCS of the P1-2 sows tended (*P* = 0.072) to be higher than those of the P3+ sows at placement (Table 3). At weaning, P1-2 sows had lower BCS compared to P3+ sows (*P* < 0.001). The P1-2 sows had greater body condition loss (negative BCS change) between the time of placement and weaning, whereas mature sows did not lose body condition (*P* < 0.001). Inclusion levels of dietary EFA in lactation did not influence BCS at weaning (*P* = 0.668) or the change in BCS from placement to weaning (*P* = 0.602). Parity 3+ sows had a higher number of total pigs born (*P* = 0.056), stillborn pigs (*P* = 0.028) and mummies (*P* = 0.040) than P1-2 sows. The level of EFA inclusion had no impact on these variables (*P* > 0.10). Litter weights after cross-fostering (*P* = 0.010) and at weaning (*P* = 0.044) were greater for P1-2 sows compared to mature sows. There were no effects of parity group or lactation EFA inclusion levels on the number of pig weaned per litter, litter daily gain, or number of no-value pigs at weaning (*P* > 0.10).

**Table 3:** Interactive effects of supplemental essential fatty acids (EFA) by parity group during lactation on sow and litter performance

Parity group	Parity 1 and 2		Parity 3+			*P-*values		
Lactation EFA inclusion	Low	High	Low	High	SEM	Parity group	EFA	Parity × EFA
Sows, n	71	69	84	85				
Lactation length, d	21.7	21.0	21.1	20.7	1.02	0.066	0.020	0.465
Feed intake, kg/d	5.2	5.2	5.5	5.5	0.14	0.027	0.596	0.952
Sow BCS								
Placement	12.91	12.68	12.41	12.44	0.24	0.072	0.585	0.569
Weaning	11.19	11.26	12.55	12.41	0.35	< 0.001	0.668	0.702
Change	−1.75	−1.37	0.17	0.04	0.29	< 0.001	0.602	0.289
Litter size, n								
Total pigs born	15.23	15.44	16.06	16.09	0.41	0.056	0.843	0.673
Pigs born alive	13.79	14.07	14.18	14.31	0.38	0.477	0.604	0.795
Stillborn pigs	1.12	1.33	1.54	1.36	0.16	0.028	0.826	0.874
Mummies	0.21	0.19	0.38	0.34	0.08	0.040	0.254	0.516
Light pigs born^a^	1.03	1.41	1.17	1.27	0.22	0.777	0.174	0.382
Weaned	11.01	10.68	10.52	10.71	0.28	0.423	0.786	0.339
No-value pigs^b^	0.54	0.51	0.63	0.82	0.21	0.126	0.598	0.509
Litter bodyweight, kg^c^								
After cross-fostering	25.9	24.6	22.0	22.2	1.38	0.010	0.612	0.480
Weaning	79.5	76.0	69.7	72.5	3.78	0.044	0.913	0.307
Daily gain, kg/d	2.96	2.83	2.84	2.91	0.12	0.367	0.975	0.223

*BCS *Body condition score

^a^ Number of pigs that were born alive and weighed 1.27 kg or less at birth. These light pigs were considered to be of lower viability and at greater risk of pre-weaning mortality, but they remained with the sow as part of the nursing litter

^b^ Number of pigs that were weaned and weighed less than 3.62 kg at weaning. These pigs were included in the number of pigs weaned

^c^ A subset of 107 sows (26, 20, 26, 28 for diet (Low and High EFA) within parity group P1 and 2 and P3+ , respectively) was used to measure litter performance

### Subsequent reproduction

**Fig. 1:**
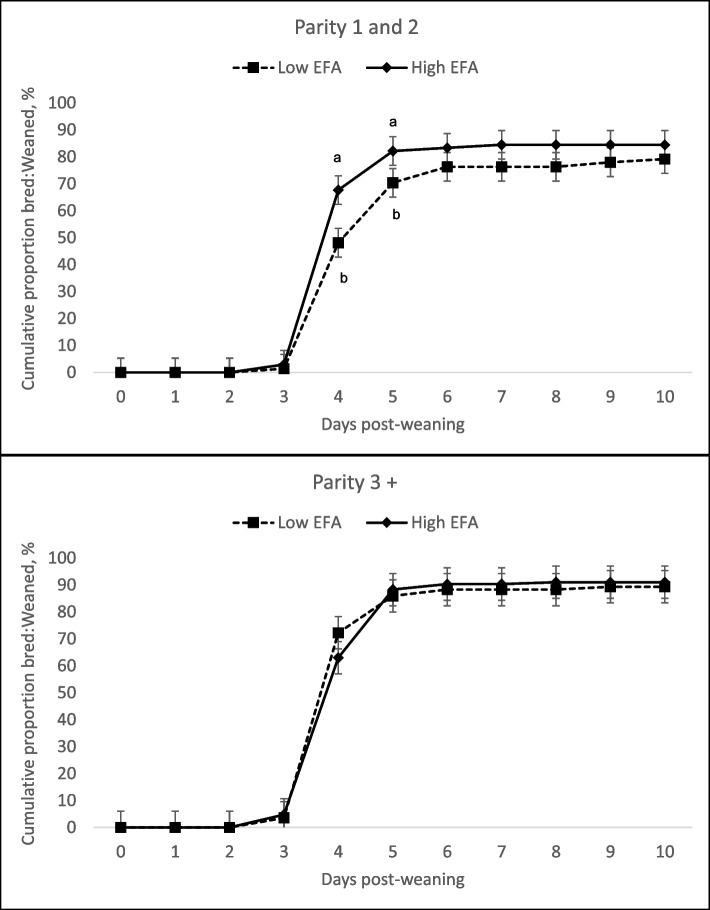
Effect of low and high dietary EFA levels fed to lactating sows on the cumulative proportion of sows bred:sows weaned. Supplemental EFA in lactation increased the proportion of P1-2 sows bred:weaned within 4 d (*P* = 0.013, SEM = 5.90) after weaning. There was no impact on P3+ sows. Estimated proportions represented by symbols without a common letter are different (*P* < 0.05)

**Fig. 2:**
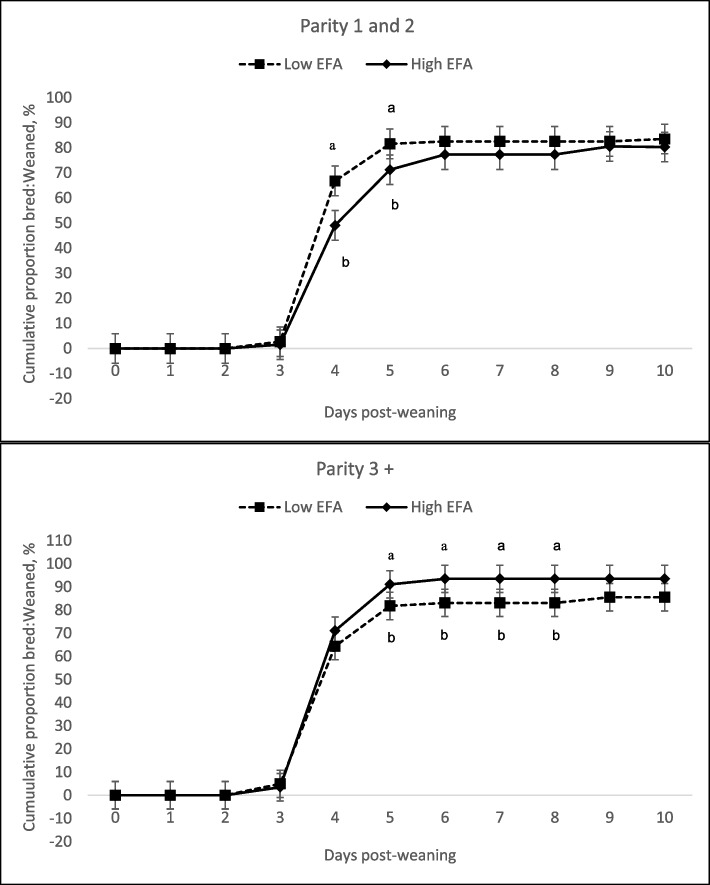
Effect of low and high dietary EFA levels fed to sows during the wean-to-breeding peiod on the cumulative proportion of sows bred:sows weaned. Feeding the high EFA diet during the wean-to-breeding period reduced the proportion of P1-2 sows bred within 4 d after weaning (*P* = 0.035, SEM = 5.90) with no effect on P3+ sows. The proportion of P3+ sows bred within 5 d after weaning increased (*P* = 0.028, SEM = 3.71), but no impact on P1-2 sows, when fed the high EFA wean-to-breeding diet. Estimated proportions represented by letters without a common letter are different (*P* < 0.05)

There were no significant three-way interactions (*P* > 0.10) and the interaction between feeding EFA in lactation and the wean-to-breeding period was not significant for any of the measured variables. Young sows (P1-2) consuming high levels of EFA during lactation displayed shorter WEI (two-way interaction, *P* = 0.035), but this was not the case in P3+ sows (Table 4). The number of sows bred by 4 d after weaning was greater for P1-2 sows that were fed high levels of EFA in lactation but not P3+ sows (two-way interaction, *P* = 0.013) (Fig. 1). A tendency for a two-way interaction (*P* = 0.087) was detected in P1-2 sows consuming high levels of EFA during the wean-to-breeding period showing an increase in the WEI in P1-2 females while P3+ sows were not impacted. Consumption of high EFA levels during the wean-to-breeding period reduced the percentage of P1-2 sows bred by d 4 and 5 d after weaning (two-way interaction, *P* < 0.040). A greater number of P3+ sows were bred by d 5 post-weaning compared to the P1-2 sows (*P* = 0.026). Inclusion of high levels of EFA in the wean-to-breeding diet increased the number of P3+ sows bred by d 5 post-weaning (two-way interaction, *P* = 0.028) (Fig. 2).

**Table 4:** Interactive effects of EFA supplementation during lactation or the following wean-to-breed period on sow subsequent reproductive performance and litter size pigs born index (total number of pigs born per 100 weaned sows)

Parity group	Parity 1 and 2				Parity 3+							
Lactation EFA	Low		High		Low		High			*P*-values^a^		
Wean-to-breed EFA	Low	High	Low	High	Low	High	Low	High	SEM	Parity group	Lactation EFA	Wean-to-breed EFA
Sows weaned, n	30	33	31	32	42	38	42	38				
Sows bred:weaned, %	91.0	84.6	97.1	87.3	91.9	95.1	91.9	97.6	2.9	0.328	0.295	0.852
WEI, d^b,c^	4.4	4.8	4.1	4.3	4.2	4.2	4.3	4.3	0.13	0.131	0.123	0.338
Bred within 4 d:weaned, %^d,e^	56.1	40.3	76.1	58.0	69.7	74.6	58.7	67.2	7.3	0.112	0.430	0.383
Bred within 5 d:weaned, %^f^	71.1	69.7	88.9	72.7	82.5	88.6	81.0	93.0	3.9	0.026	0.704	0.189
Sows farrowed:bred, %^g,h^	90.1	65.1	84.9	77.9	65.7	80.9	91.7	90.7	7.2	0.435	0.064	0.375
Removed:weaned, %^i,j^	8.2	33.1	10.7	24.8	32.1	18.7	12.2	6.7	4.1	0.740	0.070	0.328
Subsequent litter, n												
Total pig born	16.4	17.4	15.8	15.5	15.3	15.5	15.0	14.8	0.69	0.018	0.072	0.726
Pigs born alive	15.0	16.0	13.8	14.6	13.2	13.8	13.6	13.4	0.57	0.004	0.154	0.259
Stillborn pigs	1.2	1.4	1.8	0.8	1.7	1.2	1.3	1.1	0.27	0.856	0.545	0.082
Mummies^k,l^	0.2	0.1	0.3	0.2	0.3	0.4	0.1	0.2	0.09	0.477	0.414	0.697
Pigs born index, n/100 sows weaned												
Farrowing rate, %^m^	80.2	54.1	82.1	69.3	61.2	78.6	84.6	88.7	5.11	0.190	0.027	0.528
Total pigs born^n^	1,335	930	1,300	1,053	914	1,194	1,280	1,294	123.6	0.861	0.109	0.303
Pigs born alive^o^	975	602	892	740	560	865	932	958	115.4	0.830	0.062	0.652

*WEI* Wean-to-estrus interval

^a^The 3-way interaction and the interaction between EFA supplementation in lactation and EFA supplementation wean-to-breeding were not significant

^b^Two-way interaction: Parity group × Lactation EFA inclusion (*P* = 0.035)

^c^Two-way interaction: Parity group × Wean-to-breed EFA inclusion (*P* = 0.087)

^d^Two-way interaction: Parity group × Lactation EFA inclusion (*P* = 0.013)

^e^Two-way interaction: Parity group × Wean-to-breed EFA inclusion (*P* = 0.035)

^f^Two-way interaction: Parity group × Wean-to-breed EFA inclusion (*P* = 0.028)

^g^Two-way interaction: Parity group × Lactation EFA inclusion (*P* = 0.081)

^h^Two-way interaction: Parity group × Wean-to-breed EFA inclusion (*P* = 0.042)

^i^Two-way interaction: Parity group × Lactation EFA inclusion (*P* = 0.099)

^j^Two-way interaction: Parity group × Wean-to-breed EFA inclusion (*P* = 0.003)

^k^Two-way interaction: Parity group × Lactation EFA inclusion (*P* = 0.078)

^l^Two-way interaction: Parity group × Wean-to-breed EFA inclusion (*P* = 0.040)

^m^Two-way interaction: Parity group × Wean-to-breed EFA inclusion (*P* = 0.005)

^n^Two-way interaction: Parity group × Wean-to-breed EFA inclusion (*P* = 0.007)

^o^Two-way interaction: Parity group × Wean-to-breed EFA inclusion (*P* = 0.020)

**Fig. 3:**
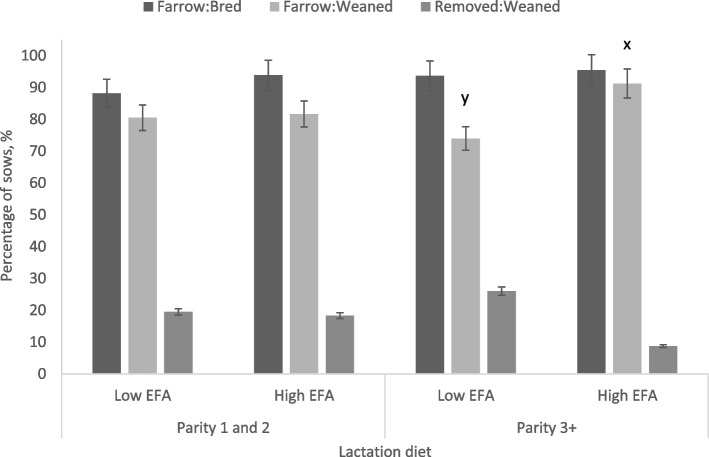
Subsequent conception and farrowing rates of weaned sows fed diets supplemented with EFA in lactation between P1-2 sows and P3+ sows. A tendency for an interaction between dietary EFA concentrations in lactation and parity group (*P* = 0.081) showed an increase in the subsequent farrowing rates of P3+ sows consuming high EFA concentrations in lactation without an effect on P1-2 sows. Means represented by bars without a common letter are different (*P* < 0.10)

**Fig. 4:**
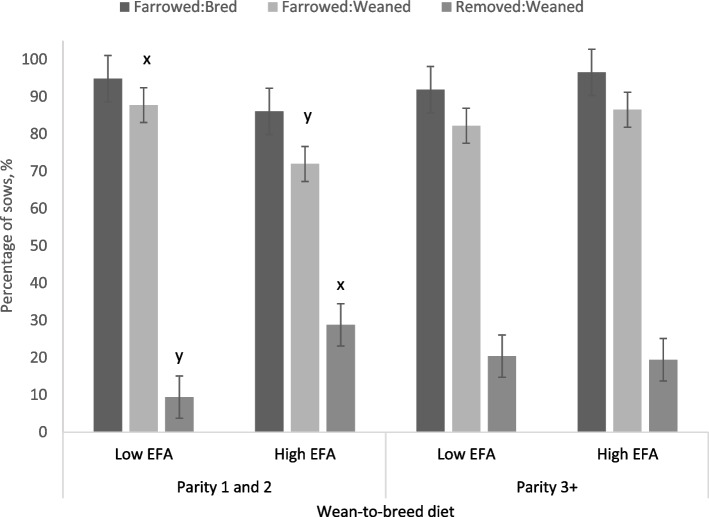
Subsequent conception and farrowing rates of weaned sows fed diets low or high inclusion of EFA during the wean-to-breed period between P1-2 sows and P3+ sows. An interaction between parity group and EFA inclusion during the wean-to-breeding period (*P* = 0.054) tended to reduce the subsequent farrowing rates of P1-2 sows but not for P3+ sows. The rate of removal for sows that were weaned was also greater in P1-2 sows that consumed high EFA levels during the post-weaning period, with no effect on P3+ sows (*P* = 0.003). Means represented by bars without a common letter are different (*P* < 0.10)

High inclusion of EFA in lactation tended to result in a higher pregnancy retention rate (sows farrowed:bred) in the subsequent gestation (*P* = 0.064) and tended to reduce the rate of removal (*P* = 0.070) (Table 4). Parity 3 and older sows fed the high EFA lactation diet tended to have a higher farrowing rate relative to sows bred (two-way interaction, *P* = 0.081) (Fig. 3). For the wean-to-breeding period, high EFA intake reduced the farrowing rate for P1-2 sows that were bred (two-way interaction, *P* = 0.042) and increased the rate of removal (two-way interaction, *P* = 0.003), but this was not the case for P3+ sows (Fig. 4).

### Subsequent litter size

Younger (P1-2) sows had a greater number of total pigs born (*P* = 0.018) and pigs born alive (*P* = 0.004) in the subsequent litter compared to mature sows (Table 4). Sows fed the high EFA lactation diet tended to have a lower number of total pigs born in the subsequent litter (*P* = 0.072). The subsequent number of stillborn pigs tended to be lower for sows receiving the high EFA diet during the wean-to-breeding period (*P* = 0.082). The number of mummies was increased for P3+ sows fed the high EFA diet during the wean-to-breeding period but did not affect P1-2 sows (two-way interaction, *P* = 0.040).

### Pig index per 100 sows weaned

Three-factor interactions between parity group, EFA inclusion, and supplementation period were not detected (*P* > 0.10) for subsequent farrowing rate or the pigs born index (Table 4). Consumption of high EFA diets during lactation increased the subsequent farrowing rate of sows weaned (*P* = 0.027) and tended to increase the number of pigs born alive per 100 sows weaned (*P* = 0.062). High EFA in wean-to-breeding diets fed to P1-2 sows reduced the subsequent farrowing rate of weaned females (two-way interaction, *P* = 0.005) but did not impact P3+ sows. As a result, the total number of pigs born and the number of pigs born alive and per 100 sows weaned was reduced in P1-2 sows when consuming high EFA levels during the wean-to-breeding period (two-way interaction, *P* < 0.050).

## Discussion

The value of a breeding female in a swine herd is based on her reproductive performance, including the total number pigs born, number of pigs born alive, number and weight of viable pigs weaned, the ability to quickly resume estrus for subsequent breeding [3], and the capacity to maintain pregnancy. Increasing the intake of EFA (linoleic and α-linolenic acid) by lactating sows has been demonstrated to be beneficial for sow subsequent reproductive performance [5, 6]. To our knowledge, there have been no published studies in which sows were fed diets containing high concentrations of EFA throughout lactation and into subsequent gestation, except for Roszkos et al. [21]. These authors explored the utilization of solely the n-3 α-linolenic acid rather than both α-linolenic and linoleic acid in combination. In the current study, we evaluated the impact of feeding both of these EFA during lactation on the subsequent reproduction of sows and further explored the impact of feeding weaned sows high levels of EFA during the weaning-to-breeding period on their subsequent reproduction.

Most of the variables that were collected during lactation were influenced more by differences in parity (P1-2 vs. P3+) than dietary EFA; the only variable that differed between the low and high EFA diets during lactation was the shorter number of days lactating, which was 0.5 d shorter for the sows consuming the high EFA diet. This difference was relatively small and using lactation length as a covariate was not significant, verifying the lack of impact of lactation length on outcome variables. Total number of pigs born per litter during the first farrowing between the EFA treatment groups was not different as expected considering that litter size is already determined in early in gestation [22] and is most unlikely impacted by dietary treatments that are initiated during late gestation or prior to farrowing. In young sows, low feed intake and nursing heavier litters will result in greater mobilization of bodily reserves, which is expected to negatively influence subsequent reproduction [1, 23]. Of the subset of litters that were weighed during the first lactation, the heavier litter weaning weights observed in the P1-2 sows compared to the P3+ sows is most likely explained by the younger sows having heavier litters following cross-fostering. This aligns with the observations seen in the current study with P1-2 sows that had a greater loss in BCS in lactation before returning to estrus and being bred.

Litter weaning weight and daily litter gain for the subset of litters did not differ between the control sows and sows fed the high EFA in lactation, which supports the findings of Rosero et al. [5]. In a study by Holen et al. [24], total litter gain and average litter gain were improved in litters that were nursing sows fed corn-soybean meal diets supplemented with either 3% soybean oil, 3% choice white grease or a combination of 3% soybean oil and 2% choice white grease compared to sows that did not receive supplemental lipids in lactation. Increased lipid supplementation during lactation has been demonstrated to increase litter gain due to the extra net energy intake [23]. In the present study, both lactation diets were supplemented with lipids at the same level (tallow or soybean oil) and contained close to the same level of net energy (calculated difference of approximately 20 kcal/kg of feed), thus no impact on milk production and litter gain was expected.

The lack of differences in litter performance from sows fed either the low or high EFA diet might be due to lactation performance and milk output being already optimized with addition of supplemental lipids in lactation diets. Milk production takes physiological priority during lactation. Inclusion of fats or oils at varying levels (ranging from 2% to 10%) increased average daily energy intake for the sow, resulting in improved litter gain as well as allowing the sow to rely less on body tissue reserves [23, 25–27]. Increased litter weights are most likely related to increased milk fat output [28] as milk production is proportionate to caloric intake [29]. Even when housed under high ambient temperatures (32 °C), milk fat output greatly increased (90 g/d) compared to thermo-neutral conditions (20 °C; 60 g/d) in sows supplemented with fat [30].

In the current study, young sows (P1-2) consuming the high EFA diet during lactation displayed a shorter WEI that was reduced by 0.37 d. Inclusion of supplemental EFA in lactation tended to increase pregnancy retention in the subsequent gestation and reduced the rate of sow removals. The dose response study by Rosero et al. [5] demonstrated that increasing linoleic acid in lactation showed a greater impact on subsequent reproductive performance in mature sows (parity 3–5) compared to primiparous sows that displayed minimal effects, except for removal (culling) rates. In that study, increasing linoleic acid intake (> 115 g linoleic acid/d) in lactation correlated with a higher number of sows being weaned, a greater proportion of sows returning to estrus, and increased pregnancy retention rates. In the same study, inclusion of at least 0.45% α-linolenic acid in conjunction with increased linoleic acid resulted in a higher proportion of sows returning to estrus and being bred (94.2% weaned:bred) and maintaining the highest percentage of pregnancies (98% pregnant:bred). Control sows fed low levels of both EFA (< 2.1 linoleic acid and < 0.15% α-linolenic acid) had higher culling rates and reduced subsequent farrowing rates [5]. van Wettere [6] showed that high linoleic acid concentration in the lactation diet (2.55%) resulted in a higher portion of sows returning to estrus within 2 weeks post-breeding compared to sows consuming levels of linoleic acid of less than 1.85% during winter months. Contrary to these results, Holen et al. [24] did not detect differences in WEI or the proportion of sows bred that subsequently farrowed when a corn-soybean meal control diet was supplemented with 3% soybean oil in the prior lactation. These authors used sows ranging from parities 2 to 9 and did not analyze effects between young and mature sows.

For subsequent litter size, supplementation of 3 g of fish oil/kg (increased intake of eicosapentaenoic [C20:5n3] and docosahexaenoic [C22:6n3] acid [metabolic derivatives of C18:3n3]) in lactation diets resulted in an increased total number of pigs born and number of pigs born alive in the subsequent litter of parity 0 to 7 sows [4]. Rosero et al. [5] showed that total number of pigs born in the subsequent litter increased in sows consuming > 115 g linoleic acid/d in the preceding lactation. Subsequent litter size, however, appeared to not be affected in response to the varying α-linolenic acid levels. In the findings from van Wettere [6], an intake of 125 g linoleic acid/d resulted in a decreased number of pigs born dead, a tendency for reduced number of mummies in the subsequent litters and, ultimately, increased number of pigs born in the subsequent litter calculated per 100 sows weaned. In the present study, subsequent number of total pigs born or number of pigs born alive did not differ in sows fed elevated concentrations of EFA in the prior lactation. When evaluating the total number of pigs born per 100 sows weaned, a greater retention of sows fed high EFA diet in the preceding lactation contributed to a 12.7% increase in the number of pigs born alive per 100 weaned sows. Calculated EFA intake for P1-2 sows consuming the low EFA diet was approximately 53 g/d and 0.5 g/d of linoleic and α-linolenic acid, respectively, whereas parity 3+ sows consumed very similar levels of these EFA (56 g/d and 0.6 g/d for linoleic and α-linolenic acid, respectively). For sows fed the high EFA diet in lactation, the calculated intake for P1-2 sows was 156 g/d and 19 g/d of linoleic and α-linolenic acid, respectively, while P3+ sows consumed 165 g/d and 20 g/d. Low EFA intake during lactation as was observed in the present study is expected to cause a negative balance of EFA because secretion of EFA in milk far surpasses EFA intake, which ultimately erodes subsequent reproductive performance [5, 11] and can be corrected by EFA supplementation [13]. As shown in the present study, increased consumption of EFA during lactation may not benefit subsequent litter sizes but more so promote better sow longevity and total output. The number of young sows consuming high EFA post-weaning that were bred by d 4 (49.1%) and d 5 (71.1%) was substantially lower than P3+ sows (71.2% at d 4 and 91.1% at d 5 post-weaning). The poor subsequent reproduction of P1-2 sows consuming high EFA in post-weaning diets resulted in elevated rates of pregnancy losses and removals. A recent study by Roszkos et al. [21] compared supplementation of fish oil (source of C20:5n3 and C22:3n3) to soybean oil (which is roughly fifteen times higher in linoleic than α-linoleic acid) throughout lactation and up to d 30 of subsequent gestation and reported that the only benefit identified was that n-3 supplemented sows had a shortened interval to estrus (by 1.4 d).

The n-6 fatty acid C18:2n6 and the n-3 fatty acid C18:3n3 are metabolic precursors to dynamic eicosanoids (lipids ≥ 20 carbons in chain length) such as prostanoids and other metabolites that are key regulators of reproduction. Polyunsaturated fatty acids accumulated by oocytes are directly linked to cell phospholipid membrane fluidity [31] and intracellular signaling mediators [32]. While the established role of endometrial prostaglandin F_2α_ is luteolytic [33], it also has a role during lactational anestrous by acting on the hypothalamic-pituitary axis to stimulate the release of luteinizing hormone that is normally suppressed in response to intense suckling [34]. Prostaglandin E_2_ plays a role in ovulation, fertilization, embryo development and early implantation in combination with prostaglandin F_2α_ [35]. Essential fatty acids are also secreted through milk for consumption by the neonatal pig to stimulate growth. Entering a negative EFA balance from inadequate dietary intake of EFA and mobilization of tissue EFA [11] puts the sow at risk of compromised subsequent reproductive performance because the reserve of EFA precursors needed are drained.

Beneficial impacts of supplemental EFA on reproductive mechanisms have been better investigated in bovine compared to porcine species due to polyunsaturated fatty acids being subjected to microbial biohydrogenation in the rumen. Consumption of rumen-inert polyunsaturated fatty acids led to increased pregnancy rates in cattle [16]. Interestingly, recipient cattle supplemented with rumen-inert essential PUFA also had increased number of successful embryo transfers [16]. Increased concentrations of n-3 polyunsaturated fatty acids in luteal tissue reduced arachidonic luteal content and inhibited intraluteal synthesis of prostaglandin F_2α_ that is detrimental to the production of luteal progesterone needed for successful pregnancy [36]. The n-6 metabolite eicosadienoic acid (C20:2n6) has the capability of acting in the endometrium and conceptus and downregulating PGF_2α_ [37]. Two other prostanoids originating from n-6 metabolites, prostaglandins E_2_ and I_2_, are involved in elongation of the conceptus, which increases the probability of pregnancy establishment [38].

A negative energy balance in lactation can compromise successful pregnancy in the subsequent breeding period as seen with prolonged return-to-breeding interval. Previous studies suggested that bodyweight loss > 10% resulted in reduced farrowing rate (number of successful pregnancies relative to the number of animals bred) and total number of pigs born in subsequent litters across all parities [1]. The highest number of losses (25%) occurred in early gestation while the conceptus is still in the embryonic phase (prior to d 35 of gestation) [39]. While ovulation rate was not reduced, embryo survival at d 28 of gestation was reduced in sows that were feed restricted in lactation [40, 41].

## Conclusions

Inclusion of high concentrations of EFA (3.0% linoleic acid and 0.38% α-linolenic acid) in lactation diets increased the proportion of pregnancy retentions throughout the subsequent gestation and reduced the percentage of sows removed from the herd, regardless of parity group. Young sows had the additional benefit of shortened wean-to-service interval when consuming high concentrations of essential fatty acids in lactation. Extending the dietary supplementation of high EFA from post-weaning until breeding appeared to be beneficial for mature sows (P3+), but not for young females (P1-2). Young sows supplemented with EFA in the post-weaning period displayed an increased number of days to service, decreased pregnancy retention throughout the subsequent gestation, and increased removal rate. These results can be strategically applied in commercial production by feeding dietary lipid sources with high concentrations of EFA during lactation for young and mature sows, and during the weaning-to-breeding period for mature sows to improve reproductive performance. Further studies need to investigate the underlying metabolic differences between the young and mature sows to explain the discrepancies.

## Data Availability

The data supporting the study findings are available from the authors upon reasonable request.

## Abbreviations

EFA: Essential fatty acids
ME: Metabolizable energy
P1-2: Parity 1 and 2 sows
P3+: Parity 3 to 9 sows
WEI: Wean-to-estrus interval
